# Community pharmacists improving equitable access to contraceptive methods: a commentary

**DOI:** 10.1007/s11096-025-01870-x

**Published:** 2025-02-21

**Authors:** Zaynah Zureen Ali, Anisa Rojanapenkul Assifi, Helen Skouteris, Stephanie Pirotta, Safeera Yasmeen Hussainy

**Affiliations:** 1 Health and Social Care Unit, School of Public Health and Preventive Medicine, Monash University, Melbourne, VIC 3004 Australia; 2 Department of General Practice, School of Public Health and Preventive Medicine, Monash University, Melbourne, VIC 3004 Australia; 3 Pharmacy Department, Peter MacCallum Cancer Centre, Melbourne, VIC 3000 Australia; 4 Sir Peter MacCallum Department of Oncology, University of Melbourne, Melbourne, VIC 3000 Australia

**Keywords:** Contraceptive methods, Community pharmacist, Prescribing, Sexual and reproductive health, Scope of practice

## Abstract

Sexual and reproductive health is an important aspect of a woman’s health and her ability to access safe, effective, affordable, and acceptable forms of fertility regulation, including contraception. This commentary piece introduces the barriers and facilitators to community pharmacists’ practising in sexual and reproductive health, and compares and contrasts interventions taking place internationally, compared to key clinical trials occurring in Australia on an expanded scope of community pharmacy practice, in prescribing the oral contraceptive pill (OCP). Modelling the practices already occurring in other high-income countries, Australia is striving to remove barriers and improve accessibility to contraceptive care, by enabling community pharmacists to prescribe contraception.

## Background

Sexual and reproductive health is vital for women and girls as it is not merely a state with the absence of disease, but relates to an individual's physical, emotional, and social well-being across all life-stages [1]. Equitable access to reliable safe, effective, and affordable high-quality sexual and reproductive health remains an ongoing area for improvement globally [1]. Over the last 10 years, Canada, New Zealand, the United States of America (USA), and the United Kingdom (UK) have commenced supporting community pharmacists to prescribe contraceptive methods, especially the OCP, enabling pharmacists to work beyond their scope of practice. This shift in access to sexual and reproductive healthcare continues to grow internationally, where an analysis in 2015 demonstrated that only 47 of 147 countries required a prescription to obtain contraceptive methods [2]. Recently, Australia has been focusing on reforming sexual and reproductive healthcare, acknowledging that this is a practice already occurring internationally [3]. New South Wales (NSW) is the only state or territory in Australia with legislation to support pharmacist prescribing of contraceptive methods (e.g. the OCP, the most commonly used contraceptive method in Australia [4]). Pharmacists are known to have extensive competencies, knowledge, and skills to support the safe use of medicines, provide medicines information to patients and other healthcare professionals, and provide reproductive health services such as contraceptive care [5]. This commentary piece introduces the barriers and facilitators to community pharmacists’ practising in sexual and reproductive health, and compares and contrasts interventions taking place internationally, compared to key clinical trials occurring in Australia on an expanded scope of community pharmacy practice, in prescribing the OCP [6].

Contraception provides women with the choice and autonomy to control their reproductive health, and allows them to play an important role in their family planning journey [7]; making access vitally important. In February 2024, the International Pharmaceutical Federation (FIP) endorsed a consensus statement: "*Ensuring access to reproductive health supplies and information, is key to people’s wellbeing and gender equality. Scientific advances provide individuals with an increasingly wide range of effective and safe contraceptive methods. Up-to-date counselling based on rights and on evidence is key to translate the increasing supply to increasing contraception choices *[8]*.”* Nonetheless, barriers still exist preventing women from being able to access equitable sexual and reproductive health information and services. In Australia, these include: inadequate rebates to pharmacists; regulatory restrictions limiting their role; inadequate community awareness of contraceptive methods; inadequate practitioner training; rurality and limited care in these areas, particularly for culturally and linguistically diverse (CALD) communities and First Nations communities; and limited contraceptive methods available on the Pharmaceutical Benefits Scheme (PBS) [9].

## Expanded scope of community pharmacy practice

Community pharmacy scope-of-practice is defined by the Pharmaceutical Society of Australia (PSA) as *‘a time-sensitive, dynamic aspect of practice that indicates those professional activities that a pharmacist is educated, competent and authorised to perform, and for which they are accountable’* [10]. Expanding pharmacists' scope-of-practice allows them to undertake roles and take on responsibilities beyond their current standard of practice [6]. It is agreed that community pharmacist-prescribing of the OCP addresses a long-standing public health issue of increasing equitable access to hormonal contraception (especially for those in rural and remote areas), allowing pharmacists to practice at a higher level, and to advocate for sexual and reproductive health services [11]. It is an emerging service that is innovative and future-focused because it considers expanding the scope of community pharmacy practice to include non-traditional practices i.e., prescribing [5].

Findings from a recently published scoping review identified successful trials where community pharmacists’ upskilled through implementation support activities were able to work to an expanded scope of practice and improve patient outcomes, however evidence specific to sexual and reproductive health is limited [6]. The review found 12 of 14 randomised controlled trials (RCTs) incorporated highly effective interventions to support community pharmacists to provide care that went beyond their usual scope of practice; only one RCT focused on delivering sexual and reproductive health services [6, 12]. By focusing specifically on Australian state-based trials examining pharmacist prescribing or resupply of the OCP, these learnings can be used to highlight similarities and differences between international and Australian pharmacy contexts in the quest to expand the scope of community pharmacy practice.

## Pharmacist prescribing practices internationally

Table 1 synthesises the evidence on community pharmacists’ prescribing practices internationally, from high-, middle-, and low-income countries, of contraceptive methods. Many pharmacists practise in jurisdictions that allow pharmacist prescribing and/or resupply of contraceptive methods, recognising that this will greatly improve equitable access and timely sexual and reproductive healthcare. This is critical as the estimated worldwide unintended pregnancy rate is 40% [13].

**Table 1:** Pharmacist prescribing practices internationally of contraceptive methods

Countries	Description
Canada	Since 2020, four provinces (Alberta, Saskatchewan, Quebec, Nova Scotia) have expanded community pharmacists' role allowing them to prescribe OCPs as well as hormonal contraceptives that do not require daily administration e.g. vaginal rings, transdermal patches, and medroxyprogesterone acetate (a progestin-containing depot) [14].
United Kingdom (UK)	The Pharmacy Contraception Service (PCS) was developed to offer people greater choice when considering whether to start or continue their current form of contraception. From 1 December 2023, the PCS enabled patients to request an OCP prescription for the *first time,* directly from their local community pharmacist.
New Zealand	Community pharmacists can resupply selected OCPs since 2021, but first-time users and women aged less than 16 years are excluded [15].
United States of America (USA)	Fifteen states introduced legislation in 2019 to authorise pharmacists prescribing the OCP [16].
Switzerland	The European Contraception Policy Atlas supports Switzerland to legislate the availability of contraceptive methods without prescriptions to reduce barriers [17]. Although, opposition to this type of service has been raised [18]. Safety concerns about contraceptive methods stem from the risk of venous thromboembolism (VTE) associated with the combined oral contraceptive pill, however, such adverse events are rare in women of reproductive age and benefits of contraception continue to outweigh risks in most women [19, 20]. Pharmacists can dispense the OCP in justified cases without a valid prescription, however the initiation of the OCP, changing between different contraceptive methods, substances, or dosages, are not currently supported [20]. In 2019, the Swiss government introduced new legislation to simplify the access of certain prescription-only items [20]. Under this new legislation, there will be a review of prescription-only items and pharmacists providing access to the OCP may be a new measure [20].
South Africa	Community pharmacists have traditionally provided primary healthcare services to those who could afford to pay [21], including family planning. South Africa recently implemented a new qualification that enables community pharmacists to deliver expanded services [21]. This means that graduates with the new qualification, as part of the expanded services, will be able to examine, diagnose, prescribe, and monitor the treatment of their patients according to the Primary Health Care Standard Treatment Guidelines of South Africa [21]. Specifically, the Primary Care Drug Therapy qualification [22] has been developed by the South African Pharmacy Council to expand and improve prescribing services [21]. This supplementary training is offered as a series of three short courses, and is funded by the pharmacists themselves [22]. Once qualified with postgraduate training, pharmacists can: prescribe medications and provide screening services in an approved primary healthcare setting, which caters for comprehensive patient history taking; physical examination (excluding internal and external genitourinary examination); Assessment of diagnosed and undiagnosed conditions listed in the Primary Health Care Standard Treatment Guidelines and Essential Medicines List; Ordering, conducting, and interpretation of diagnostic and laboratory testing; Decision on safe and appropriate therapy; Prescribing of medicines for the conditions identified for the purposes of the Primary Care Drug Therapy Qualification as per the Primary Health Care Standard Treatment Guidelines and Essential Medicine List published by the National Department of Health; monitoring of outcomes of therapy; and Referral to another healthcare provider where necessary [22]. A “specialist pharmacist” register has been maintained by the South African Pharmacy Council to indicate which particular pharmacists have a prescribing permit [22]. The Standard Treatment Guidelines has been developed alongside an Essential Medicines List, for different levels of care, primarily to *“achieve Universal Health Coverage including financial risk protection, access to quality essential health-care services, and access to safe, effective, quality, and affordable essential medicines and vaccines for all* [23]*.”* As per the National Drug Policy of 1996, the family planning stream of medicines as outlined by the Standard Treatment Guidelines, and the Essential Medicines List include: Levonorgestrel intrauterine device (52 mg); Copper intrauterine device; Progestin-only pills; Combined oral contraceptive pill; Emergency contraception; Etonorgestrel implant; and Medroxyprogesterone acetate intramuscular injection (150 mg). The Standard Treatment Guidelines and Essential Medicines List are regularly under review and must be consulted before supplying any agent. The main purpose of expanding the community pharmacy scope of practice by extending prescribing to pharmacists is to improve patient care without compromising patient safety, increase access to healthcare services, make better use of pharmacists’ skills, and harness a more flexible approach to working in primary healthcare settings [22]. A small percentage of pharmacists have completed postgraduate training to afford a prescribing authority, however not many studies have been conducted to understand the impact on healthcare [22].
United Arab Emirates	Prescribing is not a well-established service. Currently, pharmaceutical care and ‘extended’ roles are not practiced optimally, or at all. However, since 2005, pharmacy practice in Abu Dhabi has undergone several changes, namely, the reclassification of several medicines from prescription-only medicines to pharmacist-only medicines. As a result, there are regular ‘surprise’ inspections of community pharmacists, causing them to ensure that all rules are strictly followed. For example, in some emirates the OCP and antibiotics can occasionally be supplied by pharmacists without a prescription – a type of service that would rarely occur in Abu Dhabi [24].
India	A Doctor of Pharmacy program is offered as a two-year course, and this training is in conjunction to undergraduate pharmacy studies [25]. This program is intended to advance pharmacy knowledge and clinical practice skills [25]. Although, in 2015, the Pharmacy Council of India clarified that pharmacy centres were not permitted to *“diagnose disease and prescribe medicines* [25]*.”* For this reason, pharmacists with a Doctor of Pharmacy would have minimum contact with patients, and this is an area of growing contention [25].
Ghana	Unlike in high-income countries, to accommodate the lack of community pharmacists and the low presence of community pharmacies outside big cities in Ghana (such as the Greater Accra region), the Pharmacy Council of Ghana has authorised the opening of chemical shops by people who are not pharmacists, that is, licensed chemical sellers (LCSs) [26]. To be qualified as an LCS, a person must at minimum pass secondary school-level education, with basic knowledge of healthcare [27]. Pre-licensing training is mandatory, and this license is renewed yearly [27]. The LCSs are authorised to sell over-the-counter medicines to communities that the Pharmacy Council considers poor [27]. These medicines include analgesics and antimalarials, and the only antibiotic allowed is co-trimoxazole [27]. Community shops form the bulk of basic medicine supply due to lack of resources, funding, education, and facilities to accommodate more community pharmacists [27]. This highlights a major gap in healthcare but demonstrates the measures necessary for a variant pharmacy service to help Ghanian communities, even if they are not community pharmacists. This also illustrates the potential for LCSs to prescribe contraceptive methods, whilst appreciating that the pharmacy profession is still developing in Ghana to meet the growing needs of their population.

Australia is on its way to achieving the same, and this movement in pharmacy practice will create fairer opportunities for women to take control and remain in-charge of their contraceptive choice.

## Continued dispensing vs OCP resupply/prescribing in Australia

Community pharmacists in Australia have already demonstrated competency in supplying prescription-only medication, through the continued dispensing arrangement [28]. In Australia, the ‘*Pharmaceutical Benefits Scheme (PBS)’* lists all medicines available to be dispensed at a government-subsidised price. Continued dispensing allows pharmacists to supply the PBS maximum quantity of prescription-only medicine, to a person under specific circumstances. This is exclusive to essential medicines for the management of chronic diseases including asthma, heart disease, dyslipidaemia, diabetes, and since 1 July 2022, contraception. This can only be claimed once within 12 months. It is often actioned where there is an immediate need for the medicine but the prescriber is unable to be contacted and/or is unable to provide a PBS prescription at the time [28]. Whilst there may be some overlap with continued dispensing, the resupply/prescribing initiative in Australia is a standalone service that provides patients with an opportunity to seek approved contraceptive methods from their local community pharmacist, without a prescription from a GP. This empowers pharmacists with an expanded scope of practice and provides patients with equitable and enhanced access to contraception. Moreover, continued dispensing only applies to levonorgestrel, levonorgestrel with ethinylestradiol, norethisterone, and norethisterone with ethinylestradiol [29]. Depending on the state/territory, Australian community pharmacists can supply a wider range of contraceptive methods in the resupply/prescribing service.

## Pharmacist prescribing in Australia

Australia commenced implementation of the resupply and prescribing of contraception by community pharmacists in October 2023. This aligns with the Australian *2023 Senate Inquiry into Universal Access to Reproductive Healthcare* recommendation that health professionals working in sexual and reproductive health should work to their full scope of practice in a clinically safe and effective way. By extension, this acknowledges the *‘Unleashing the Potential of our Health Workforce—Scope of Practice Review’* [4], by outlining the practical improvements needed to support greater productivity and improve safe, equitable, and affordable pharmacy services.

Several resupply and prescribing trials are currently underway across four states and one territory in Australia (New South Wales, Victoria, Australian Capital Territory, South Australia, Tasmania and Queensland). By allowing community pharmacists to resupply and/or prescribe the OCP, the goal of these trials is to address barriers to access by removing appointment waiting times, and appointment costs [9]. The NSW Government-sponsored Clinical Trial: Extended Supply of Oral Contraceptive Pills by Community Pharmacists, a non-randomised trial (PATH-OC), is currently underway in community pharmacies in NSW and the Australian Capital Territory (ACT) [30]. Here, we reflect on the PATH-OC trial using publicly accessible trial information based on the protocol (and not the PATH-OC trial results), with mention of similar work conducted in other Australian states and territories. PATH-OC will implement and evaluate community pharmacists’ resupply of OCPs to women aged 18 to 35 years. In this context, the term ‘resupply’ relates to community pharmacists being able to provide the OCP without a prescription from the GP, where the patient successfully meets the criteria [30]. Since writing this commentary, the PATH-OC trial has generated such strong evidence that as of 28 September 2024, pharmacists in NSW who have completed the training and work in community pharmacies with suitable facilities will be able to resupply the OCP as part of standard care [3]. This follows the successful facilitation of the first phase of the NSW Pharmacy Trial in May 2024, which recorded more than 3,300 pharmacists in NSW delivering more than 18,000 consultations to women aged 18 to 65 with symptoms of an uncomplicated urinary tract infection (UTI) [3].

As a requirement set out in the PATH-OC trial protocol, pharmacists must have completed online training before implementing the intervention [30]. This is a common practice that ensures pharmacists are upskilled and trained to deliver a service not previously offered [6]. Further, ‘structured prescribing’ forms a large portion of pharmacist prescribing internationally, and is the main intervention in the PATH-OC trial, where community pharmacists are authorised to prescribe OCPs. Whilst the results of the PATH-OC trial have not yet been published, the international literature reports positive patient outcomes when a community pharmacist is able to prescribe, such as improved medication adherence [6]. This is a critical outcome for OCP-taking as improved adherence reduces the risk of unwanted pregnancy, experienced by 1 in 4 women in Australia [31]. Furthermore, increased uptake in referrals by community pharmacists has the potential to facilitate more timely and efficient diagnosis of primary health conditions [6]. In the PATH-OC trial, participating pharmacists were reimbursed $20 AUD per consultation to recognise their time, expertise, and training expectations when delivering the intervention [30]. This addresses service delivery gaps commonly reported by community pharmacists, where they do not feel valued or appreciated for their services [6]. The PATH-OC intervention required the use of a private consultation room, which gives patients a sense of comfort when consulting with their pharmacist and may result in successful outcomes [6].

A novel aspect of the PATH-OC intervention was the requirement that pharmacists complete intervention support activities in the form of university-developed modules about their legal and ethical obligations, particularly where an expanded scope of community pharmacy practice is identified [6, 30]. It is especially important in the PATH-OC trial as pharmacists need to understand the difference between usual care (regular dispensing of OCP) and interventional care. Unique to the PATH-OC trial, the pharmacy consultation and structured prescribing was guided by a tailored IT program providing recommendations from the Australian Therapeutic Guidelines [30]. It safeguards the practitioner and consumer from medication misadventure, because care is underpinned by current best practice. Hence, unlike the trials in the scoping review, the implementation strategy design of the PATH-OC trial provides ongoing support through practice change facilitators visiting community pharmacies during the study [30]. This type of support may help pharmacists at the time they are delivering the intervention.

In the Victorian Community Pharmacist Statewide Pilot, South Australia Community Pharmacy Oral Contraceptive Pill Resupply Services trial, and Tasmania Community Pharmacy Program, pharmacists are required to inform the patient’s regular GP of the consultation e.g. resupply of the OCP [32–34]. Where subsequent consent is provided, pharmacists will be required to update the patient’s electronic health record with the specific type of OCP supplied. This ensures that care is transparent between all healthcare professionals, and facilitates collaborative monitoring [32, 33]. In Western Australia however, it is mandated that the electronic health record be updated. In South Australia and Western Australia, pharmacists must complete cultural safety and gender diversity training so that the resupply service offered is equitable, which addresses barriers women from CALD backgrounds may experience when accessing services [9, 33, 35]. Queensland is the only state that through its contraception pilot allows pharmacists to prescribe, in addition to the OCP, the combined hormonal vaginal ring, the progestogen-only contraceptive pill, and depot medroxyprogesterone acetate (DMPA). This offers women choice, particularly where they may have a contraindication to any oestrogen-components or derivatives, prefer a more discrete form of contraception, or have poor medication adherence [36].

## Conclusion

Modelling the practices already occurring in other high-income countries, Australia is striving to remove barriers and improve accessibility to contraceptive care, by enabling community pharmacists to prescribe contraception. The findings from the international literature exploring interventions implemented to expand a community pharmacy scope of practice and the PATH-OC trial, reveal that understanding the similarities and differences across trials world-wide, is necessary to continue to strengthen sexual and reproductive health service delivery globally. Queensland has demonstrated the most diverse expansion to community pharmacy scope of practice in the world by allowing at least four types of contraceptive methods to be prescribed by Australian community pharmacists [36]. Once available, the next steps are to consult the findings from the PATH-OC trial and similar trials in Australia, to assist the Australian Government in implementing the required policy change to ensure sustainable, equitable access to sexual and reproductive health services in community pharmacy is achievable.
